# Lower 24‐h urinary potassium excretion is associated with higher prevalent depression and anxiety status in general population

**DOI:** 10.1002/brb3.2842

**Published:** 2023-03-15

**Authors:** Zihao Wu, Mulalibieke Heizhati, Junli Hu, Mengyue Lin, Lin Gan, Mei Li, Wenbo Yang, Ling Yao, Jing Hong, Le Sun, Jing Li, Wei Li, Nanfang Li

**Affiliations:** ^1^ Hypertension Center of People's Hospital of Xinjiang Uygur Autonomous Region; Xinjiang Hypertension Institute; National Health Committee Key Laboratory of Hypertension Clinical Research; Key Laboratory of Xinjiang Uygur Autonomous Region “Hypertension Research Laboratory” Xinjiang Clinical Medical Research Center for Hypertension (Cardio‐Cerebrovascular) Diseases Urumqi 830001 China

**Keywords:** 24‐h urinary potassium excretion, anxiety status, depression status, potassium intake

## Abstract

**Background:**

Uncertainty remains about the association of potassium (K) intake with depression and anxiety status. We explored their relationship using 24‐h urinary K, reflecting K intake, in general population.

**Methods:**

We collected 24‐h urine and performed self‐rating depression and anxiety scales (SDS, SAS) cross‐sectionally in adults selected by random sampling in China. SDS and SAS standard score ≥50 defined depression and anxiety status. Participants were divided into three groups (T1, T2, and T3) by 24‐h urinary K tertile. Odds ratios (OR) and 95% confidence intervals were calculated. Sensitivity analysis was performed by excluding anti‐hypertensive agent takers.

**Results:**

546 participants comprised current analytical sample. First, T1 and T2 groups showed higher SDS scores (40.0 vs 40.0 vs 36.0, *p* = .001), prevalence (19.8 vs 15.9 vs 7.1%, *p* = .002), whereas increased adjusted odds for depression status only in T1 group (OR = 2.71, *p* = .017), compared with T3 group. Second, T1 and T2 groups showed higher SAS scores (38.0 vs 40 vs 35.0, *p* < .001) and prevalence (14.8 vs 21.4 vs 8.8%, *p* = .003), whereas increased adjusted odds for anxiety status only in T2 group (OR = 2.07, *p* = .042), compared with T3 groups. Third, T1 and T2 groups showed higher prevalence (10.4% vs 11.5% vs 2.7%, *p* = .004) and adjusted odds (OR = 3.71, *p* = .013; OR = 3.66, *p* = .014) for co‐existent anxiety and depression status, compared with T3 group. Most results remained consistent in sensitivity analysis.

**Conclusions:**

Lower K intake is implicated in presence of anxiety and depression status in general population; this may provide basis for programs to increase K intake and prevent disease.

## Graphical abstract

We classified dietary potassium intake with 24‐h urine collection, found a independent association between potassium intake and depression and anxiety status.

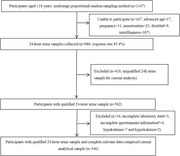

## 1 INTRODUCTION

Mental disorders have become one of the serious public health problems in the world, with depression and anxiety as the two most common mental disorders in the general medical setting (Andrade et al., 2003; Kroenke et al., 2007). Studies show that the lifetime prevalence of depression and anxiety averaged 11.2% and 3.7%, respectively (Kessler et al., 2015; Ruscio et al., 2017). Since the 1990s, depressive disorders, second only to ischemic heart disease, have become the second greatest contributor to global disease burden, quantified as years of life lived in less than ideal health (Vos et al., 2012). In addition, disability‐adjusted life‐years of depression and anxiety increased by 14.3% and 12.8% during 2007−2017 (GBD 2017 Dalys & Hale Collaborators, 2018). Therefore, exploring modifiable risk factors is necessary to prevent depression and anxiety.

Although accumulating studies have been conducted in the past few decades, the etiologies for depression and anxiety are not understood clearly. Female gender, low socioeconomic status, less social support, stress, alcohol and drug abuse, genetic and epigenetic factors, dysregulation of gut microbiota, and several disease conditions contribute to increased risk for depression and anxiety (Hosseinzadeh et al., 2016). In addition, balanced diet or dietary patterns play important roles in human model of thinking and human behavior, as the intake of foods affects human cognition, memory capacity, and emotions (Huang et al., 2019); balanced dietary patterns such as the Mediterranean diet have been uniquely associated with a lower risk of depression or depressive symptoms (Parletta et al., 2019; Rienks et al., 2013). Besides a balanced diet, isolated nutrients are another element that might also be involved in mental disorders. Emerging evidence suggests that potassium (K) intake might be one of the modifiable risk factors for depression and anxiety, whereas inconclusive. In 1423 Japanese elderly population, K intake is significantly and negatively correlated with depressive symptoms among female participants (Thi Thu Nguyen et al., 2019). Furthermore, K intake is found lower in 59 patients with depression aged ≥18 years in another study (Kaner et al., 2015) and serum K is decreased slightly in 200 preoperative patients with anxiety (McCleane & Watters, 1990). Moreover, consumption of vegetables and fruits, well‐known as sources of K and recommended for the prevention of depression (Opie et al., 2017), is negatively correlated with severity of depressive symptoms (Mamplekou et al., 2010) and presence of anxiety (Sadeghi et al., 2021; Saghafian et al., 2018). Importantly, an intervention study reports that a low‐sodium and high‐K diet seems to have positive effects on general mood state including assessment of depression and anxiety (Mrug et al., 2019; Torres et al., 2008). Therefore, it is reasonable to speculate that K intake is associated with the development of depression and anxiety and a proper K intake may have positive effects on the prevention/reduction of the disease status, if the association between the two can be established.

Overall, the associations between K and depression and anxiety have been evaluated with premature status in previous studies and not without limitations (Faghih et al., 2020; Kaner et al., 2015; Karkishchenko & Khaĭtin, 1983; Mamplekou et al., 2010; McCleane & Watters, 1990; Mrug et al., 2019; Opie et al., 2017; Sadeghi et al., 2021; Saghafian et al., 2018; Shakya et al., 2021; Thi Thu Nguyen et al., 2019; Torres & Nowson, 2012; Torres et al., 2008; Yannakoulia et al., 2008). Some studies were conducted in selected population (patients (McCleane & Watters, 1990; Torres & Nowson, 2012), single gender (Torres & Nowson, 2012), the elderly (Thi Thu Nguyen et al., 2019), or the young (Faghih et al., 2020; Mrug et al., 2019), and in small sample (Kaner et al., 2015; Torres et al., 2008), and some failed to use suggested methods for estimation of K intake (questionnaires (Kaner et al., 2015; Karkishchenko & Khaĭtin, 1983; McCleane & Watters, 1990; Sadeghi et al., 2021; Shakya et al., 2021; Thi Thu Nguyen et al., 2019), or 12‐hour urinary K excretion (Mrug et al., 2019). It has been reported that 77% of K ingested is excreted through urine, which makes 24‐h urine sample collection as the gold standard to objectively measure K intake (World Health Organization, 2012). Therefore, we explored the association of K, using 24‐h urinary K (24‐h UK) excretion, a recommended method for K intake assessment, and depression and anxiety status in Chinese adults from general population.

## 2 MATERIALS AND METHODS

### 2.1 Study population

**FIGURE 1:**
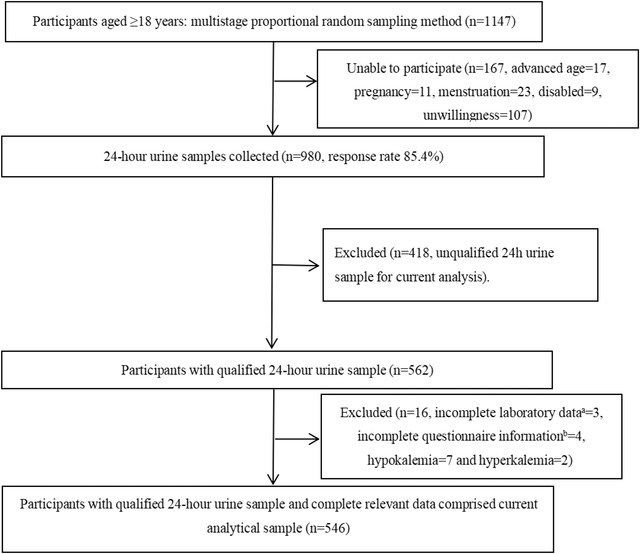
Flowchart for study participants. (a) Laboratory data include serum potassium test value, 24‐h urinary potassium test value, and 24‐h urinary sodium test value. (b) Questionnaire information included general information, SAS data, SDS data, and PSQI data.

In this cross‐sectional study, we obtained study participants aged ≥18 years using multistage proportional random sampling method from Emin county, Xinjiang, China between March and June 2019. Eligible populations were asked to participate in 24‐h urine collection and post examination. Details were described in our previous study (Abudoureyimu et al., 2021; Li et al., 2022; Wang et al., 2021). In brief, the county was divided into 20 sites at the first stage. At the second stage, 10 sites were selected. At the third stage, participants were selected from locals, based on inclusion and exclusion criteria as in Figure 1.

### 2.2 Measures

#### 2.2.1 24‐h urine sample collection and measurement

As described previously (Abudoureyimu et al., 2021; Wang et al., 2021), a complete scheme was applied to the collection and detection process of 24‐h urine samples. Urine samples meeting any of the following exclusion criteria were not included in this study: the urine volume < 500 ml; the duration of specimen collection < 20 h; reported more than 100 ml of urine lost during collection; the 24‐h urinary creatinine (24‐h UCr) per kilogram of body weight was not up to standard (Mohammadifard et al., 2019; Nerbass et al., 2014). The detection of electrolytes (i.e., K, sodium, etc.) and creatinine in urine samples were carried out uniformly.

#### 2.2.2 Data and blood sample collection

Data were collected on participants’ demographic characteristics (age, gender), socioeconomic status (occupation, education), lifestyles (cigarette and alcohol consumption), sleep quality using Pittsburgh sleep quality index (PSQI), depression and anxiety status using self‐rating depression scale (SDS) and self‐rating anxiety scale (SAS), and medical histories (hypertension, diabetes, dyslipidemia, and relevant therapeutic agents). Trained investigators also measured body weight, height, and blood pressure (BP), according to protocol.

Body weight was accurate to 0.1 kg. Height was measured to the nearest 0.1 cm. BP was expressed as the average value of three measurements of the electronic sphygmomanometer. Body mass index (BMI) was calculated by dividing body weight by the square of height (kg/m^2^).

Venous blood samples were obtained after an overnight fasting and serum creatinine, lipid profiles, and glucose were measured at People's hospital of Tacheng on the same day of collection, to where driving takes 40 min.

#### 2.2.3 SDS and SAS scales, depression, and anxiety status

The SDS is a self‐report scale, which contains 20 items reflecting subjective feelings of depression, of which 10 are positive and 10 are negative. Each item was rated with respect to how participants felt using a four‐point Likert‐type scale. Options include: 1  =  little or no time, 2  =  a small part of the time, 3  =  quite a lot of time, 4  =  most or all of the time. Forward scoring questions were scored as 1, 2, 3, and 4; reverse scoring questions were scored as 4, 3, 2, and 1. Reverse scoring question number: 2, 5, 6, 11, 12, 14, 16, 17, 18, and 20. The original total score of SDS is between 20 and 80, and the result is usually expressed as the standard total score of SDS, which is obtained by multiplying the original score by 1.25 and taking the integer part (Zung, 1965).

The SAS is also a self‐report scale with 20 items covering a series of anxiety symptoms (Zung, 1971). In the current study, participants were instructed to choose how often they experienced each symptom over the past week given on a four‐point Likert‐type scale, ranging from 1 (little or no time) to 4 (most or all of the time). Items include positive and negative experiences. Forward scoring questions were scored as 1, 2, 3, and 4; reverse scoring questions were scored as 4, 3, 2, and 1. Reverse scoring question number: 5, 9, 13, 17, and 19. The standard total score of the SAS ranges from 25 to 100. The higher the standard score, the more serious the symptom.

Depression status was defined as SDS standard score ≥50, anxiety status as SAS standard score ≥50, and coexistence of the two as SDS standard score ≥50 and SAS standard score ≥50 (Zung et al., 1990).

#### 2.2.4 Definitions of other covariates

Hypertension: systolic BP ≥140 mmHg and or diastolic BP ≥90 mmHg and or antihypertensive agent intake within the previous 2 weeks of survey (Joint committee for guideline revision, 2019). Diabetes mellitus: fasting blood glucose (FBG) ≥ 7.0 mmol/L and or self‐reported previous diagnosis by clinicians and or intake of hypoglycemic agents within the past 2 weeks of survey (Alberti & Zimmet, 1998). Dyslipidemia: triglyceride (TG) ≥2.3 mmol/L and or total cholesterol (TC) ≥6.2 mmol/L and or high density lipoprotein cholesterol < 1.0 mmol/L and or low density lipoprotein cholesterol ≥4.1 mmol/L and or having received lipid‐lowering treatment during the past 2 weeks (Joint committee for guideline revision, 2018). Poor sleep quality was defined as a PSQI score > 6 (Antza et al., 2018; Buysse et al., 1989; Zheng et al., 2013). Education attainment status was categorized as middle school and lower and high school and higher. Occupations were divided into two types as mental and manual work. Alcohol intake was defined as drinking at least once a week within 1 month at the survey time point (Wang et al., 2018). Cigarette consumption was defined as smoking more than 20 packs of cigarettes and smoking currently (Wang et al., 2018).

### 2.3 Statistical analysis

Participants were divided into three groups by the tertile of 24‐h UK as T1, T2, and T3 groups. Continuous variables including age, BMI, BP, 24‐h UNa, and 24‐h UK were presented as means ± standard deviations and were analyzed using ANOVA test if normally distributed; otherwise, presented as median and 25th–75th percentiles and analyzed by Mann–Whitney U test or Kruskal–Wallis H test. Categorical variables were displayed in the form of frequency (n) and proportion (%) and examined through Chi‐square test.

Logistic regression analysis was used to assess the association of the tertiles of 24‐h UK (T3 group of 24‐h UK as the reference) and the presence of depression status, anxiety status, and co‐existence of the two, and results were presented as unadjusted and adjusted odds ratios (ORs) and the 95% confidence intervals (CI).

Independent variables significantly relevant to depression and or anxiety status (*p* < .1) in univariate Logistic regression analysis were adjusted in multivariate logistic analysis. Tolerance and the variance inflation factor were examined to identify multicollinearity, which could be concerned if the variance inflation factor was > 10 and the tolerance was < 0.10.

Sensitivity analysis was conducted to compare SDS, SAS score, and logistic regression analysis by excluding participants with hypertension under anti‐hypertensive treatment.

Results were considered statistically significant if two‐tailed *p* value was less than .05. All analyses were performed with SPSS statistical software, version 20.0 (Chicago, IL, USA).

## 3 RESULTS

### 3.1 Participant characteristics at baseline

As in Figure 1, in total, 1147 adults aged ≥18 years were randomly selected and asked to participate, of whom 980 agreed to participate with a response rate of 85.4%. Among 980 participants, 562 participants provided complete 24‐h urine sample. Finally, 546 participants with complete data on 24‐h urine and relevant parameters comprised current analytical sample.

### 3.2 General information of participants

In current analytical participants, median age was 48.2 (36.2, 55.7) years and 57.9% were women (Table 1). Participants were tertiled into three grous as T1 (< 970.9 mg), T2 (970.9–1337.8 mg), and T3 (≥1337.8 mg) groups by 24‐h UK.

**TABLE 1:** Characteristics of total study participants and by tertile of 24‐h urinary potassium excretion

Characteristics	Total	T1 (< 970.9 mg)	T2 (970.9 to 1337.8 mg)	T3 (≥1337.8 mg)	X^2^, Z/P
N	546	182	182	182	
Age (years)	48.2 (36.2, 55.7)	45.8 (32.2, 54.0)	48.2 (34.8, 55.4)	49.8 (42.9, 56.6)	13.001/0.002
Gender (women, *n*, %)	316 (57.9)	109 (59.9)	120 (65.9)	87 (47.8)	12.703/0.002
Education (≥high school, *n*, %)	244 (44.7)	77 (42.3)	91 (50.0)	76 (41.8)	3.121/0.210
Occupation (mental worker, *n*, %)	202 (37.0)	65 (35.7)	76 (41.8)	61 (33.5)	2.839/0.242
Cigarette consumption (*n*, %)	101 (18.5)	40 (22.0)	35 (19.2)	26 (14.3)	3.662/0.160
Alcohol intake (*n*, %)	130 (23.8)	32 (17.6)	39 (21.4)	59 (32.4)	11.871/0.003
Hypertension (*n*, %)	197 (36.1)	61 (33.5)	64 (35.2)	72 (39.6)	1.538/0.464
Anti‐hypertensive agents (*n*, %)	105 (19.2)	29 (15.9)	43 (23.6)	33 (18.1)	3.672/0.159
Systolic blood pressure (mmHg)	125.2 (115.3, 138.8)	122.3 (114.5, 136.8)	125.5 (115.3, 138.7)	126.8 (116.6, 141.0)	3.491/0.175
Diastolic blood pressure (mmHg)	79.5 (73.0, 88.0)	79.9 ± 11.9	80.6 ± 11.2	80.5 (74.3, 88.2)	1.808/0.405
Diabetes (*n*, %)	52 (9.5)	10 (5.5)	17 (9.3)	25 (13.7)	7.171/0.028
Fasting blood glucose (mmol/L)	5.11 (4.60, 5.82)	5.0 (4.5, 5.6)	5.1 (4.7, 5.8)	5.2 (4.7, 6.1)	5.433/0.066
Dyslipidemia (*n*, %)	181 (33.2)	51 (28.0)	58 (31.9)	72 (39.6)	5.659/0.059
Serum total cholesterol (mmol/L)	4.53 (3.81, 5.22)	4.5 ± 1.1	4.5 (3.8, 5.2)	4.6 (3.9, 5.4)	1.738/0.419
Serum triglyceride (mmol/L)	1.10 (0.74, 1.68)	1.0 (0.7, 1.5)	1.1 (0.7, 1.5)	1.3 (0.9, 2.0)	19.731/ < 0.001
Poor sleep quality (*n*, %)	240 (44.0)	83 (45.6)	87 (47.8)	70 (38.5)	3.518/0.172
PSQI score	5.0 (3.0, 8.0)	5.0 (3.0, 8.0)	5.0 (3.0, 9.0)	4.0 (3.0, 7.0)	4.446/0.108
Body mass index (kg/m^2^)	26.6 (23.6, 29.2)	25.6 (22.3, 28.5)	25.9 (23.4, 29.3)	27.4 (25.0, 29.8)	20.253/ < 0.001
Serum potassium (mmol/L)	4.3 (4.1, 4.6)	4.3 (4.1, 4.6)	4.3 (4.1, 4.6)	4.3 (4.1, 4.6)	1.285/0.526
24‐h urinary potassium (mg)	1133.5 (888.5, 1476.1)	772.6 (635.3, 890.0)	1133.5 (1036.2, 1224.6)	1640.9 (1475.0, 1978.3)	484.446/ < 0.001
24‐h urinary sodium (mg)	3287.7 (2351.3, 4461.5)	2602.7 (1837.0, 3780.8)	2313.7 (3312.8, 4266.7)	4238.1 (3071.2, 5341.6)	79.772/ < 0.001
24‐h urine creatinine (mmol)	8.1 (6.5, 10.2)	6.9 (5.3, 8.5)	7.9 (6.7, 9.5)	9.8 (7.8, 12.8)	102.386/ < 0.001
24‐h urine volume (ml)	1241.5 (895.8, 1571.3)	941.5 (670.0, 1313.5)	1234.0 (903.3, 1623.5)	1452.0 (1202.5, 1851.3)	94.546/ < 0.001

Abbreviations: PSQI, Pittsburgh sleep quality index; SAS, self‐rating anxiety scale.; SDS, self‐rating depression scale.

Participants in T3 and T2 groups were significantly older (49.8 vs 48.2 vs 45.8 years, *p* = .002), more alcohol takers (32.4 vs 21.4 vs 17.6%, *p* = .003), more diabetic (13.7 vs 9.3 vs 5.5%, *p* = .028), with higher TG (1.3 vs 1.1 vs 1.0 mmol/L), and higher BMI (27.4 vs 25.9 vs 25.6 kg/m^2^), compared with those in T1 group.

### 3.3 The SDS scores and depression status

As in Table 2, the median of SDS and SAS scores among the participants was 38.0 (32.0, 45.0) and 37.0 (32.0, 45.0), respectively.

**TABLE 2:** Comparison of SDS and SAS standard score among different 24‐h UK tertile groups

	Overall	T1 (< 970.9 mg)	T2 (970.9 to 1337.8 mg)	T3 (≥1337.8 mg)	*p*	*p*_1_	*p*_2_	*p*_3_
Total participants	n = 546	n = 182	n = 182	n = 182				
SDS standard score	38.0 (32.0, 45.0)	40.0 (33.0, 46.0)	40.0 (33.0, 45.0)	36.0 (31.0, 42.0)	.001	1.000	.007	.004
SAS standard score	37.0 (32.0, 45.0)	38.0 (32.0, 45.0)	40.0 (32.0, 46.2)	35.0 (30.0, 41.0)	< .001	.649	.019	< .001
Not on antihypertensive agents	*n* = 441	*n* = 147	*n* = 147	*n* = 147				
SDS standard score	38.0 (32.0, 43.0)	40.0 (33.0, 46.0)	40.0 (32.0, 45.0)	36.0 (31.0, 41.0)	.001	1.000	.001	.009
SAS standard score	37.0 (31.0, 43.0)	38.0 (32.0, 45.0)	40.0 (32.0, 45.0)	35.0 (30.0, 40.0)	.002	1.000	.021	.002

Abbreviations: SDS, Self‐rating depression scale; SAS, self‐rating anxiety scale.

*p*, among group comparison; *p*_1_, T1 versus T2; *p*_2_, T1 versus T3; *p*_3,_ T2 versus T3.

Participants in T1 (40.0 vs 36.0, *p* = .007) and T2 groups (40.0 vs 36.0, *p* = .004) showed significantly higher SDS scores than did those in T3 group, which remained consistent in sensitivity analysis by excluding hypertensives under anti‐hypertensive treatment.

Participants in T1 and T2 groups showed significantly higher prevalence of depression status in total participants (19.8 vs 15.9 vs 7.1%, *p* = .002), than did those in T3 groups, which is largely consistent in men (T1 vs T3: 16.9 vs 5.3%, *p* = .023) and in women participants (T1 vs T3: 21.9 vs 8.6%, *p* = .042) (Table 3).

**TABLE 3:** Prevalence of depression and anxiety status in total participants and in stratified participants by tertile of 24‐h UK

	Overall	T1 (< 970.9 mg)	T2 (970.9 to 1337.8 mg)	T3 (≥1337.8 mg)	*p*	*p*_1_	*p*_2_	*p*_3_
Total participants	546	182	182	182				
Depression (n, %)	78 (14.3)	36 (19.8)	29 (15.9)	13 (7.1)	.002	.885	.002	.050
Anxiety (n, %)	82 (15.0)	27 (14.8)	39 (21.4)	16 (8.8)	.003	.236	.321	.002
Depression and anxiety (n, %)	45 (8.2)	19 (10.4)	21 (11.5)	5 (2.7)	.004	1.000	.023	.007
Men	230	77	77	76				
Depression (n, %)	18 (7.8)	13 (16.9)	1 (0.1)	4 (5.3)	.001	.001	.023	1.000
Anxiety (n, %)	12 (5.2)	5 (6.5)	4 (5.2)	3 (3.9)	.779	–	–	–
Depression and anxiety (n, %)	6 (2.6)	4 (5.2)	1 (1.3)	1 (1.3)	.219	–	–	–
Women	316	105	106	105				
Depression (n, %)	60 (19.0)	23 (21.9)	28 (26.4)	9 (8.6)	.003	1.000	.042	.003
Anxiety (n, %)	70 (22.2)	22 (21.0)	35 (33.0)	13 (12.4)	.001	.105	.406	.001
Depression and anxiety (n, %)	39 (12.3)	15 (14.3)	20 (18.9)	4 (3.8)	.003	.937	.064	.003

*p*, among‐group comparison; *p*_1_, T1 versus T2; *p*_2_, T1 versus T3; *p*_3_, T2 versus T3.

As in Table 4, compared with the participants in T3 group, participants in T2 (OR = 2.46, 95%CI: 1.24, 4.91, *p* = .010) and T1 (OR = 3.21, 95%CI: 1.64,6.28, *p* = .001) groups showed elevated ORs for presence of depression status, whereas the association remained significant only for T1 group in model adjusted for age, gender, education attainment status, occupation, body mass index, systolic and diastolic blood pressure, diabetes, dyslipidemia, sleep quality, serum K, and 24‐h UNa, which showed significant association with presence of depression status in univariate logistic regression (Table S1). Furthermore, the association was more augmented in sensitivity analysis by exclusion of patients with hypertension under anti‐hypertensive treatment (ORs of T3 vs T2 vs T1: 1 vs 2.65 vs 3.90, *p* = .066 and = .010, respectively).

**TABLE 4:** Logistic regression analysis for the associations of tertiles of 24‐h UK excretion with the presence of depression and anxiety (OR, 95%CI, *p*)

	Depression status		Anxiety status		Depression and anxiety status	
	Crude model	Adjusted model	Crude model	Adjusted Model	Crude model	Adjusted model
Total participants						
24‐h UK tertiles						
T3	Ref	Ref	Ref	Ref	Ref	Ref
T1	3.21 (1.64, 6.28), 0.001	2.71 (1.20, 6.14), 0.017	1.81 (0.94, 3.48), 0.077	1.16 (0.54, 2.48), 0.710	4.13 (1.51, 11.31), 0.006	3.71 (1.31, 10.50), 0.013
T2	2.46 (1.24, 4.91), 0.010	1.61 (0.72, 3.62), 0.249	2.83 (1.52, 5.28), 0.001	2.07 (1.03, 4.18), 0.042	4.62 (1.70, 12.53), 0.003	3.66 (1.30, 10.30), 0.014
Sensitivity analyses by exclusion of antihypertensive agent takers						
T3	Ref	Ref	Ref	Ref	Ref	Ref
T1	5.36 (2.28, 12.59), < 0.001	3.90 (1.38, 11.06), 0.010	1.92 (0.91, 4.03), 0.086	1.18 (0.50, 2.77), 0.708	4.23 (1.38, 12.98), 0.012	3.99 (1.26, 12.62), 0.019
T2	3.81 (1.57, 9.24), 0.003	2.65 (0.94, 7.51), 0.066	2.38 (1.14, 4.97), 0.021	1.87 (0.84, 4.19), 0.126	4.72 (1.54, 14.48), 0.007	3.74 (1.18, 11.86), 0.025

For depression status, adjusted model was adjusted for age, gender, education attainment status, occupation, body mass index, systolic and diastolic blood pressure, diabetes, dyslipidemia, sleep quality, serum K, and 24‐h urinary sodium excretion. For anxiety status, adjusted model was adjusted for gender, education attainment status, occupation, cigarette and alcohol use, systolic and diastolic blood pressure, dyslipidemia, sleep quality, and 24‐h urinary sodium excretion. For co‐existent depression and anxiety status, adjusted model was adjusted for gender, education attainment status, occupation, systolic and diastolic blood pressure, and sleep quality.

### 3.4 The SAS score and anxiety status

Participants in T1 (38.0 vs 35.0, *p* = .019) and T2 groups (40.0 vs 35.0, *p* < .001) showed significantly higher SDS scores than did those in T3 group, which remained consistent in sensitivity analysis by excluding hypertensives under anti‐hypertensive treatment (Table 2).

Accordingly, participants in T1 (14.8% vs 8.8%, *p* = .321) and T2 (21.4% vs 8.8%, *p* = .002) groups showed higher prevalence of anxiety status in total participants, compared with those in T3 groups, whereas with statistical significance only between T2 and T3 groups, which is largely consistent in women (T2 vs T3: 33.0 vs 12.4%, *p* = .001), but not men (Table 3).

In logistic regression analysis (Table 4), compared with the reference T3 group, participants in T1 (OR = 1.81, 95%CI: 0.94, 3.48, *p* = .077) and T2 (OR = 2.83, 95%CI: 1.52,5.28, *p* = .001) groups showed marginal and significant elevation in ORs for the presence of anxiety in crude model, which remained significant only for T2 group (OR = 2.07, 95%CI: 1.03, 4.18, *p* = .042) in adjusted model for variables selected using univariate logistic regression (Table S1). The association was diminished in adjusted model of sensitivity analysis by exclusion of hypertensives under treatment.

### 3.5 Co‐existence of anxiety and depression status

As in Table 3, compared with T3 groups, participants in T1 (10.4% vs 2.7%, *p* = .023) and T2 (11.5% vs 2.7%, *p* = .007) groups showed significantly higher presence of co‐existent anxiety and depression status in total participants, which is largely consistent in women (T1 vs T2 vs T3: 14.3% vs 18.9 vs 3.8%, *p* = .003) but not in men.

In logistic regression analysis (Table 4), compared with the participants in T3 group, participants in T1 (OR = 3.71, 95%CI: 1.31, 10.50, *p* = .013) and T2 (OR = 3.66, 95%CI: 1.30, 10.30, *p* = .014) groups showed significantly higher ORs for co‐existent anxiety and depression status in the model adjusted for gender, education attainment status, occupation, BP, and sleep quality that showed higher ORs for the outcome as Table S2. Furthermore, the association remained consistent in sensitivity analysis by exclusion of hypertensives under treatment (ORs of T3 vs T2 vs T1: 1 vs 3.74 vs 3.99, *p* = .025 and = .019, respectively).

## 4 DISCUSSION

To our knowledge, current study is the first to explore the relationship between 24‐h UK excretion and depression status, anxiety status, and co‐existence of the both in relatively large sample community‐based general population. Main results showed that SDS and SAS score and prevalence of depression status, anxiety status, and co‐existence of the two are significantly higher in participants with lower 24‐h UK excretion, compared to those with higher 24‐h UK excretion. In logistic regression analysis, compared with those in the highest tertile of 24‐h UK excretion, participants in the lower tertiles showed elevated odds for presence of depression status, anxiety status, and their co‐existence, which remained consistent in sensitivity analysis by excluding potential confounding of anti‐hypertensive agents for depression status and for co‐existence of the both. These results imply that lower K intake, indicated by lower UK excretion, may be involved in the development of depression status and or anxiety status in the general population.

Observations from the current study add evidence on the ongoing uncertainty of K and depression and anxiety status. Current findings are consistent with results of previous reports. For example, some studies based on DASH diet model have shown that low sodium and high potassium intake can significantly reduce depression and anxiety score and improve depression and anxiety status (Mrug et al., 2019; Torres et al., 2008). In addition, the current findings may extend some previous studies from the clinical environment to the general adult population. For example, consumption of foods high in sodium and low in potassium contributes to the development of depressive and anxiety symptoms in early adolescence (Mrug et al., 2019), and low‐sodium and high‐potassium diet seems to have an overall positive effect on depressive and anxiety mood state (Torres et al., 2008).

Urinary potassium excretion has been established as a marker of overall diet quality, with higher potassium excretion positively correlated with greater intake of vegetables, fruits, whole grains, fish, and poultry, and negatively correlated with the intake of fast food and red meat (Mente et al., 2009). Therefore, with regard to public health, prevention, control, or improvement of depression, anxiety, and the comorbidity by K supplements could have public health implications since it is feasible to increase K intake by fresh vegetables and fruits, and even salt substitute, which is a practical and cost‐effective approach to supplement K and has been shown to slow the incidence of hypertension (Bernabe‐Ortiz et al., 2020), another risk factor for depression and anxiety.

In the current study, we included several confounding factors for parameters of interest. For example, we had data on anthropometric (BMI) and socioeconomic indicators (education attainment status and occupation), disease history (diabetes, hypertension), and lifestyle factors (cigarette and alcohol use and sleep quality), which have effects on interest of outcome. We also collected data on anti‐hypertensive agent use, most of which exerts effects on K and Na. Furthermore, we performed univariate and multi‐variate logistic regression and sensitivity analysis to obtain objective association of 24‐h UK and depression and/or anxiety status, although some of the data showed significant differences among groups. For example, BMI increased significantly from T1 to T3 group of UK tertile, whereas its effects on the results were ruled out using regression analysis.

The main strength of this study is the objective measurement of K excretion using 24‐h urine samples. Second, the current study was conducted in general population with wide age ranges from both genders. Therefore, results may be generalizable, although limited to a certain county. Third, we adjusted for the sleep quality, an independent risk factor for depression and anxiety (Huang & Zhu, 2020), in logistic regression analysis, and the association still remained significant. Nonetheless, the current study also contains some limitations. A key limitation is that we failed to obtain causality between K with depression and or anxiety due to the cross‐sectional nature of the study. Second, due to seasonal or daily changes in urinary potassium excretion, a single 24‐h urine sample may not reflect the usual dietary intake or pattern of participants. Nevertheless, we collected urine samples on weekdays and weekends, and from spring to summer, which may have reduced the relevant variability. In addition, we used SDS and SAS to evaluate the depressive and anxiety mood of the study population and roughly screen out possible depression and anxiety status patients. Nevertheless, SDS and SAS have been widely used including scientific researches with higher diagnostic sensitivity (SDS: 92%; SAS: 89.0%) and specificity (SDS: 77%; SAS: 69.0%) (Dunstan et al., 2017; Gabrys & Peters, 1985). In addition, both questionnaires are confirmed to be reliable and valid in Chinese population (Liu et al., 2018). Moreover, we failed to assess overall dietary intake pattern in this study population, which may provide more information on the association of K intake and depression and or anxiety. However, the main objective of the study was to assess the association of K and depression and or anxiety status, and K in 24‐h urine sample may provide more accurate assessment of 24‐h K intake, since studies show that 77% of K ingested is excreted through urine (World Health Organization, 2012).

## 5 CONCLUSIONS

Lower 24‐h UK excretion shows independent association with higher SDS and SAS scores and with higher prevalent depression and or anxiety status in general adults from China, suggesting lower K intake may be involved in depression and or anxiety, and this could lead to optimization programs focused on increasing potassium intake at the population level, which has been shown to be feasible and result in disease prevention.

## AUTHOR CONTRIBUTIONS

Study design was performed by Nanfang Li1, Mulalibieke Heizhati, and Zihao Wu. Data collection was performed by Mulalibieke Heizhati, Junli Hu, Mengyue Lin, Mei Li, Wenbo Yang, Ling Yao, Jing Hong, Le Sun, Jing Li, and Wei Li; and data analysis/interpretation was performed by Zihao Wu. Manuscript drafting or manuscript revision for important intellectual content was performed by Zihao Wu, Nanfang Li, Junli Hu, and Mulalibieke Heizhati. All authors contributed to the writing, review, and editing of the article. All authors have read and agreed to the published version of the manuscript.

## CONFLICT OF INTEREST

The authors declare no conflict of interest.

### PEER REVIEW

The peer review history for this article is available at https://publons.com/publon/10.1002/brb3.2842.

## Supporting information

Table S1 Variable selection to be adjusted using univariate logistic regression analysis and multicollinearity assessment.
Table S2 Variable selection to be adjusted using univariate logistic regression analysis and multicollinearity assessment.

## Data Availability

The data that support the findings of this study are available from the corresponding author upon reasonable request.
