# Supplementary material for: Lower 24‐h urinary potassium excretion is associated with higher prevalent depression and anxiety status in general population
Source: Brain Behav. 2023 Mar 15;13(4):e2842. doi: 10.1002/brb3.2842 (PMC10097074; doi:10.1002/brb3.2842)
Supplement: Supplementary file 1 — Table S1 Variable selection to be adjusted using univariate logistic regression analysis and multicollinearity assessment. Table S2 Variable selection to be adjusted using univariate logistic regression analysis and multicollinearity assessment. [file BRB3-13-e2842-s001.docx]

| Sup Table 1. Variable selection to be adjusted using univariate logistic regression analysis and multicollinearity assessment. | | | | | | |
| --- | --- | --- | --- | --- | --- | --- |
|  | Depression status | | | Anxiety status | | |
| Variables | OR, 95%CI, P | Tolerance | VIF | OR, 95%CI, P | Tolerance | VIF |
| 24h UK tertiles T3 | Ref | 0.93 | 1.07 | Ref | 0.91 | 1.09 |
| T1 | 3.21 (1.64, 6.28), 0.001 |  |  | 1.81 (0.94,3.48), 0.77 |  |  |
| T2 | 2.46 (1.24, 4.91), 0.010 |  |  | 2.83 (1.52,5.28), 0.001 |  |  |
| Age | 0.97 (0.95,0.99), 0.015 | 0.57 | 1.75 | 0.99 (0.97,1.01), 0.458 | / | / |
| Gender (women vs men) | 2.76 (1.58,4.81), <0.001 | 0.76 | 1.31 | 5.16 (2.72,9.79), <0.001 | 0.46 | 2.09 |
| Education (≥ vs <high school) | 2.52 (1.53,4.15), <0.001 | 0.41 | 2.44 | 2.05 (1.27,3.31), 0.003 | 0.43 | 2.43 |
| Occupation (mental vs manual) | 2.70 (1.66,4.40), <0.001 | 0.40 | 2.50 | 2.11 (1.32,3.40), 0.002 | 0.34 | 2.95 |
| Cigarette consumption ( yes vs no) | 1.45 (0.35,2.86), 0.282 | / | / | 0.31 (0.13,0.73), 0.007 | 0.66 | 1.50 |
| Alcohol intake (yes vs no) | 1.51 (0.81,2.79), 0.192 | / | / | 0.35 (0.17,0.72), 0.004 | 0.69 | 1.45 |
| Systolic blood pressure | 0.97 (0.96,0.99), 0.001 | 0.30 | 3.31 | 0.98 (0.96,0.99), 0.005 | 0.34 | 2.95 |
| Diastolic blood pressure | 0.97 (0.95,0.99), 0.005 | 0.34 | 2.91 | 0.97 (0.95,0.99), 0.007 | 0.35 | 2.86 |
| Diabetes (yes vs no) | 1.10 (0.50,2.43), 0.812 | / | / | 1.60 (0.78,3.25), 0.196 | / | / |
| Dyslipidemia (yes vs no) | 1.08 (0.65,1.79), 0.767 |  |  | 1.62 (1.00,2.62), 0.048 | 0.81 | 1.24 |
| Sleep quality (poor vs good) | 2.61 (1.59,4.30), <0.001 | 0.90 | 1.11 | 4.30 (2.55,7.26), <0.001 | 0.93 | 1.08 |
| Body mass index | 0.92 (0.87,0.98), 0.008 | 0.81 | 1.24 | 1.00 (0.95,1.05), 0.886 | / | / |
| Serum potassium | 0.53 (0.26,1.11), 0.093 | 0.93 | 1.07 | 0.90 (0.45,1.77), 0.768 | / | / |
| 24 hour urinary sodium | 0.99 (0.98,0.99), 0.009 | 0.83 | 1.37 | 0.99 (0.98,0.99), <0.001 | 0.92 | 1.09 |

| Sup Table 2. Variable selection to be adjusted using univariate logistic regression analysis and multicollinearity assessment. | | | |
| --- | --- | --- | --- |
|  | Co-existent depression and anxiety status | | |
| Variables | OR, 95%CI, P | Tolerance | VIF |
| 24h UK tertiles T3 | Ref | 0.92 | 1.09 |
| T1 | 4.13 (1.51, 11.31), 0.006 |  |  |
| T2 | 4.62 (1.70, 12.53), 0.003 |  |  |
| Age | 0.97 (0.95,0.99), 0.020 | 0.57 | 1.75 |
| Gender (women vs men) | 4.98 (1.90,13.07), <0.001 | 0.55 | 1.80 |
| Education (≥ vs <high school) | 3.35 (1.72,6.55), <0.001 | 0.42 | 2.40 |
| Occupation (mental vs manual) | 3.44 (1.81,6.50), <0.001 | 0.40 | 2.49 |
| Cigarette consumption ( yes vs no) | 0.29 (0.09,0.97), 0.044 | 0.68 | 1.46 |
| Alcohol intake (yes vs no) | 0.57 (0.25,1.30), 0.180 | / | / |
| Systolic blood pressure | 0.97 (0.95,0.99), 0.003 | 0.31 | 3.25 |
| Diastolic blood pressure | 0.97 (0.93,0.99), 0.021 | 0.90 | 1.11 |
| Diabetes (yes vs no) | 0.92 (0.32,2.68), 0.880 | / | / |
| Dyslipidemia (yes vs no) | 1.38 (0.74,2.58), 0.310 | / | / |
| Sleep quality (poor vs good) | 3.47 (1.78,6.77), <0.001 | 0.80 | 1.25 |
| Body mass index | 0.97 (0.90,1.04), 0.368 | / | / |
| Serum potassium | 0.96 (0.40,2.30), 0.923 | / | / |
| 24 hour urinary sodium | 0.99 (0.98,1.00), 0.002 | 0.90 | 1.10 |
